# Diagnostic and Prognostic Value of Soluble Syndecan-1 in Pleural Malignancies

**DOI:** 10.1155/2014/419853

**Published:** 2014-07-24

**Authors:** Filip Mundt, Ghazal Heidari-Hamedani, Gustav Nilsonne, Muzaffer Metintas, Anders Hjerpe, Katalin Dobra

**Affiliations:** ^1^Division of Pathology, Department of Laboratory Medicine, Karolinska Institutet, F46, Huddinge University Hospital, 141 86 Stockholm, Sweden; ^2^Division of Cognitive Neurophysiology and Osher Center for Integrative Medicine, Department of Clinical Neuroscience, Karolinska Institutet, 171 77 Stockholm, Sweden; ^3^Stress Research Center, Stockholm University, 106 91 Stockholm, Sweden; ^4^Department of Chest Diseases, Eskisehir Osmangazi University Medical Faculty, 260 40 Eskisehir, Turkey

## Abstract

*Background*. The distinction between malignant and benign pleural effusions is a diagnostic challenge today and measuring soluble biomarkers could add to the diagnostic accuracy. Syndecan-1 is a proteoglycan involved in various cellular functions and is cleaved from the cell surface in a regulated manner. The shed fragment, which can be recovered in effusion supernatant and in serum, retains its binding capacities, but often with different functions and signalling properties than the cell-bound form. *Aim*. This study aimed to investigate the diagnostic and prognostic value of soluble syndecan-1 in pleural effusions and sera from patients with pleural malignancies. *Study Design*. Using two cohorts of patients, we assessed the diagnostic and prognostic value of soluble syndecan-1 in pleural effusions and sera, using enzyme-linked immunosorbent assays. *Results*. In pleural effusions, syndecan-1 distinguished malignant and benign diseases, with an odds ratio of 8.59 (95% CI 3.67 to 20.09). Furthermore, syndecan-1 in pleural effusions predicted a survival difference for patients with pleural metastatic disease and malignant mesothelioma of 11.2 and 9.2 months, respectively. However, no such effects were seen when syndecan-1 was measured in serum. *Conclusion*. Soluble syndecan-1 is a promising candidate biomarker for the cytopathological diagnosis and prognostication of malignant pleural effusions.

## 1. Introduction

Pleural effusion is a common symptom of cancers in the pleural cavities. Accumulation of pleural fluid is caused partly by an increase in vascular permeability and tissue leakage and can limit lung expansion and impede breathing. The effusion is withdrawn, and malignant cells from pleural effusions are often available for cytopathological diagnosis long before biopsies are obtained [1]. Pleural effusions are in these cases often the first material available for the diagnosis [2, 3]. Tumours affecting the pleural space include metastatic adenocarcinomas from the lung (36%), breast (25%), ovary and gastric cancer (5%), and malignant lymphomas (10%) [4–6]. The primary tumour of the pleura is malignant mesothelioma; however, it is overall less frequent than metastatic cancers [6, 7].

The cytological diagnosis of malignant disease in the pleura is supported by ancillary methods such as immunocytochemistry, fluorescent in situ hybridization (FISH), electron microscopy, and biomarker analyses [8, 9]. Among proposed biomarkers, the secreted phosphoprotein osteopontin is secreted by several different tumour types [10–18]. When a definite diagnosis is not reached by these methods, clinical and radiological findings may motivate a more invasive sampling of tissue [19]. However, these invasive procedures would preferably be avoided when the diagnosis can be reached by less invasive methods [6].

Syndecan-1 (CD138), a cell surface proteoglycan, has been proposed as a cellular marker for distinguishing adenocarcinoma from mesothelioma [20–22]. This proteoglycan regulates various biological processes including cell proliferation, differentiation, invasion, migration, and angiogenesis. These processes are largely regulated by syndecan-1 through the interactions with growth factors, growth factor receptors, and several integrins [23–26]. The extracellular domain of syndecan-1 is proteolytically cleaved by metalloproteinases [27, 28] and thereby is shed to body fluids. The ratio of membrane-bound and shed syndecan-1 is altered in certain pathological conditions, including cancer and metastasis [29, 30]. However, it is noteworthy that, depending on tumour type, syndecan-1 has shown to be either a tumour suppressor or a tumour promoter [31]. The presence of syndecan-1 on mesothelioma cells is associated with favourable prognosis [32], while high levels of syndecan-1 in breast cancer indicate poor prognosis [33]. Similarly, high level of soluble syndecan-1 in serum indicates poor prognosis for patients with multiple myeloma [34] and lung cancer [35]. This dual effect of syndecan-1 seems to depend on the source and forms of syndecan-1: whether it is tumour cell-derived or synthesized by the stroma and whether it is cell surface-bound or shed.

This study aimed to evaluate the diagnostic and prognostic performance of soluble syndecan-1 in patients' serum and pleural effusions.

## 2. Materials and Methods

### 2.1. Study Participants

In this study, 256 pleural effusions (74 carcinoma, 89 malignant mesothelioma, and 93 benign effusions) and 231 serum samples (74 carcinoma, 91 malignant mesothelioma, and 66 benign conditions) were analysed for syndecan-1 and compared to their osteopontin levels. Pleural effusions were prospectively and consecutively collected in different time periods at the Department of Pathology and Cytology, Karolinska University Hospital in Huddinge, Sweden, between the years 2005 and 2011. Late time periods only included patients with lung cancer or malignant mesothelioma. Serum samples were prospectively and consecutively collected at the Chest Diseases Department of Eskisehir Osmangazi University in Eskisehir, Turkey, between the years 2002 and 2004. Serum samples were collected as part of a parallel study that evaluates the diagnostic effect of seven different biomarkers (including syndecan-1) and their combined diagnostic value for a malignant pleural mesothelioma. All samples were collected before any treatment was given. Samples were centrifuged for 1,700 g for 10 minutes without delay. Acellular supernatants were stored without additives at −20°C (effusions) or −80°C (sera). For patient demographics, see Table 1.

Inclusion criteria have been previously described [37]. In brief, all included effusions from malignant involvement of the pleura contained malignant cells. Diagnoses of malignant mesothelioma were further verified on histopathology supported by immunohistochemistry or on electron microscopy of effusion cell pellets. Metastatic pleural malignancies were diagnosed by histopathology and/or cytolopathology supported by immunocytochemistry with at least 4 antibodies. Patients diagnosed with nonmalignant disease were followed up for at least one year and excluded if they were diagnosed with any type of malignancy or were deceased, within a year from sampling. No attempt was done to distinguish exudates from transudates. Patients thought to have benign asbestos pleurisy were monitored by thoracoscopy with biopsies showing fibrinous pleuritis.

Metastatic pleural disease correlates with an advanced stage, while information of stage for malignant mesothelioma patients was not available for this study. For this reason we measured vascular endothelial growth factor (VEGF) in a subset of pleural effusions from patients with malignant mesothelioma (*n* = 16), since VEGF has been described as a surrogate marker for stage in this disease [38].

In order to evaluate the relation between cell-bound and shed syndecan-1, a separate cohort of additional 18 pleural effusions, containing well-preserved malignant cells, was collected from 2012 to 2013 at Karolinska University Hospital in Huddinge. These samples were compared simultaneously for their pleural effusion concentrations of soluble syndecan-1 and their expression of membrane-bound syndecan-1 on tumour cells.

### 2.2. Enzyme-Linked Immunosorbent Assay (ELISA)

Syndecan-1 and VEGF were measured using ELISA: Human CD138 (syndecan-1) from Gen-Probe Diaclone, France (cat. number 950.640.192) and Human VEGF Quantikine ELISA from R&D Systems, UK (cat. number DVE00), respectively. ELISAs were performed according to the manufacturer's instructions. Effusions were diluted 1 : 3 for syndecan-1 analysis and 1 : 5, 1 : 10, or 1 : 25 for VEGF analysis using kit-dilution buffers as blanks. Optical densities were determined using a spectrophotometer (BioTek's PowerWave HT, Winooski, VT, USA) at 450 nm. Patient samples were analysed in duplicates by investigators blind to patients' diagnoses and survival times.

### 2.3. Immunocytochemistry

To investigate the relationship between soluble and cell-bound syndecan-1, the latter was assessed by immunocytochemistry on tumour cells from the pleural effusion paired with their ELISA readout of shed syndecan-1 levels in the corresponding effusion supernatant. For immunocytochemistry, the pleural effusions were centrifuged for 10 min at 8,000 g and if necessary, erythrocytes were lysed using ammonium chloride (BD Pharm Lyse, BD Biosciences, CA, USA). Cells were immobilized on SuperFrost Plus Slides (Thermo Fisher Scientific Inc., Waltham, MA, USA), using cytospin preparations. Immunostaining was performed using a Lecia BOND-III automated IHC. Epitope retrieval was done by pretreating the slides in a citrate buffer, pH 6.0 (Bond Epitope Retrieval Solution 1, Leica Microsystems GmbH). Endogenous peroxidase activity was blocked with 3% H_2_O_2_ followed by incubation with syndecan-1 primary antibody (CD138, clone MI15, diluted 1 : 100, IgG1, DakoCytomation, CA, USA). Bound antibodies were demonstrated with the Bond Polymer Refined Detection kit (Leica DS 9800), and the cells were counterstained with haematoxylin.

Two experienced cytopathologists (KD and AH) evaluated all slides independently and were blinded to clinical diagnosis and levels of soluble syndecan-1. Cell-bound syndecan-1 expression was assessed by semiquantitative scoring which includes (i) the percentage of syndecan-1 positive tumour cells (0–100%) and (ii) the signal intensity (4-point scale). The scoring for signal intensity corresponded to 0, negative; 1, weak staining; 2, moderate positive; and 3, strong positive staining. The semiquantitative immunocytochemical (ICC) score for the cell-bound syndecan-1 expression level was provided by the multiplication of the percentage (0–100%) of syndecan-1 positive staining by the factor (1–4) corresponding to the staining intensity of the tumour cells.

### 2.4. Statistical Analyses 

#### 2.4.1. Analyses of Biomarker Expression in Pleural Effusions and Sera

Levels of soluble syndecan-1 and osteopontin were compared between patients with cancer and those without, using the Mann-Whitney test calculating two-tailed exact *P* values. Nonparametric tests were used since biomarkers were not normally distributed (D'Agostino and Pearson omnibus normality test; data not shown). Analyses of syndecan-1 levels between multiple patient subgroups were performed using the nonparametric Kruskal-Wallis one-way analysis, with Dunn's post hoc test comparing mean rank of each patient's group to the mean rank of the benign patients' group. Correlation between soluble syndecan-1 in paired effusion and serum samples was analysed using Spearman correlation. Osteopontin levels were extracted from an earlier study [37] and used as a reference biomarker for malignancy [10–18]. Correlation between soluble syndecan-1 and osteopontin levels, in either pleural effusions or sera, was analysed using Spearman correlation. Statistical analyses were performed using GraphPad Prism software (v. 6.01, GraphPad Software Inc.).

#### 2.4.2. Logistic Regression

Logistic regression was used to create a predictive model for each biomarker, with cancer or without cancer as outcome, coded as 1 and 0, respectively. Univariate odds ratios were calculated. Model calibration is recommended by several studies [39, 40]. Model calibration was assessed by graphical inspection as well as calculation of Nagelkerke's *r* ^2^. Goodness of fit was quantified using the le Cessie-van Houwelingen-Copas-Hosmer unweighted sum of squares test [41] using the R software v. 3.0.1.

#### 2.4.3. Receiver Operator Characteristic (ROC) Analyses

ROC plots with areas under the curves (AUC) and their 95% confidence intervals were made with the GraphPad Prism software. The calculations of sensitivity, specificity, positive predictive value, and negative predictive value were calculated with cutoffs from maximum sensitivity × specificity from ROC curves.

#### 2.4.4. Discrimination Slopes

Discrimination slopes are recommended by several studies [39, 40]. Discrimination plots were constructed to assess the predicted probability for each biomarker of a malignant pleural effusion. Discrimination slope (DS) is the difference between predicted probabilities; in this study it is the probability that syndecan-1 or osteopontin predicts malignant effusions over a benign. Analyses were performed using the R software v. 3.0.1 and visualized using GraphPad Prism software.

#### 2.4.5. Survival Analysis

Survival analysis was performed with cutoff values based on the highest and most significant hazard ratio [42]. Survival times were available for a subset of patients; thus, survival data for patients providing serum samples was available for 19 patients, which is too few to extract a hazard ratio based cutoffs. So, for comparisons in sera the median was instead used as a dichotomizing value. With determined cutoffs, the prognostic information was estimated using the Kaplan-Meier survival analysis. The log-rank (Mantel-Cox) test compared survival curves and estimated hazard ratios and *P* values. Survival analyses were performed and graphs were created using the GraphPad Prism software.

#### 2.4.6. Correlation between Soluble and Cell-Bound Syndecan-1 Levels in Paired Pleural Effusion and Cytospin Samples

Spearman correlation analysis was used to assess the relationship between soluble syndecan-1 in effusions and corresponding cell-bound syndecan-1 in patients suffering from various pleura malignancies. Analyses were performed and graphs were created using the GraphPad Prism software.

### 2.5. Ethical Permits

This study was approved by the ethical review board of Stockholm, Sweden (2009/1138-31/3), and the ethical review board of Eskisehir University, Turkey. All patients had given informed consent.

## 3. Results

### 3.1. Expression Levels of Syndecan-1 and Osteopontin

**Figure 1:**
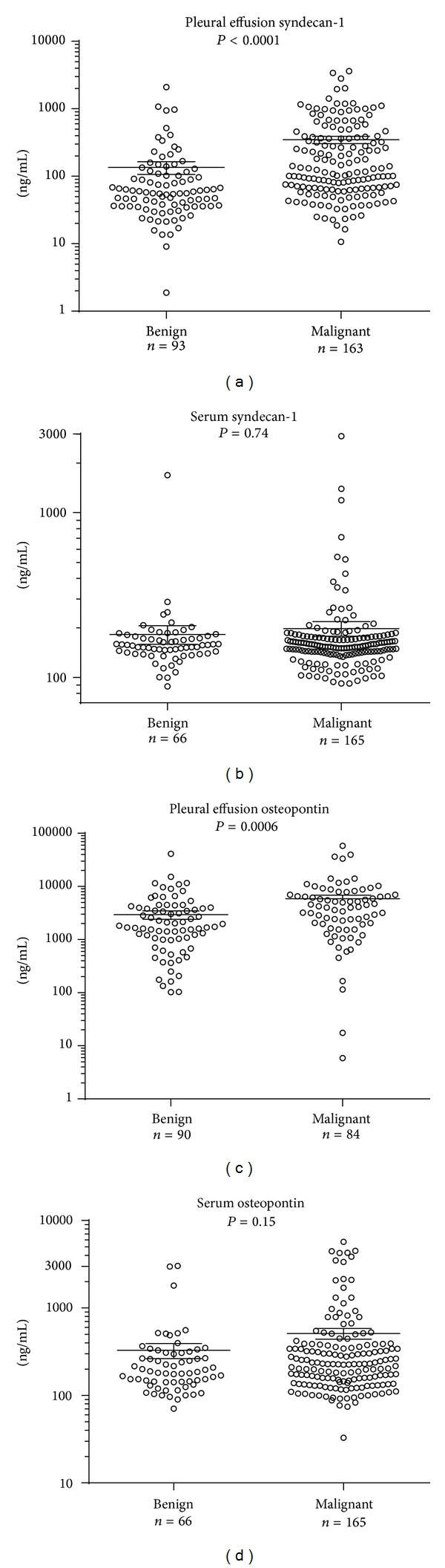
Levels of syndecan-1 and osteopontin are higher in pleural effusions from malignant tumours than from benign conditions. Syndecan-1 and osteopontin expression levels in benign and malignant pleural effusions, as measured by ELISA. *P* values are from Mann-Whitney tests, with Dunn's* post hoc* tests. Lines represent mean and error bars represent standard error of the mean (SEM).

**Figure 2:**
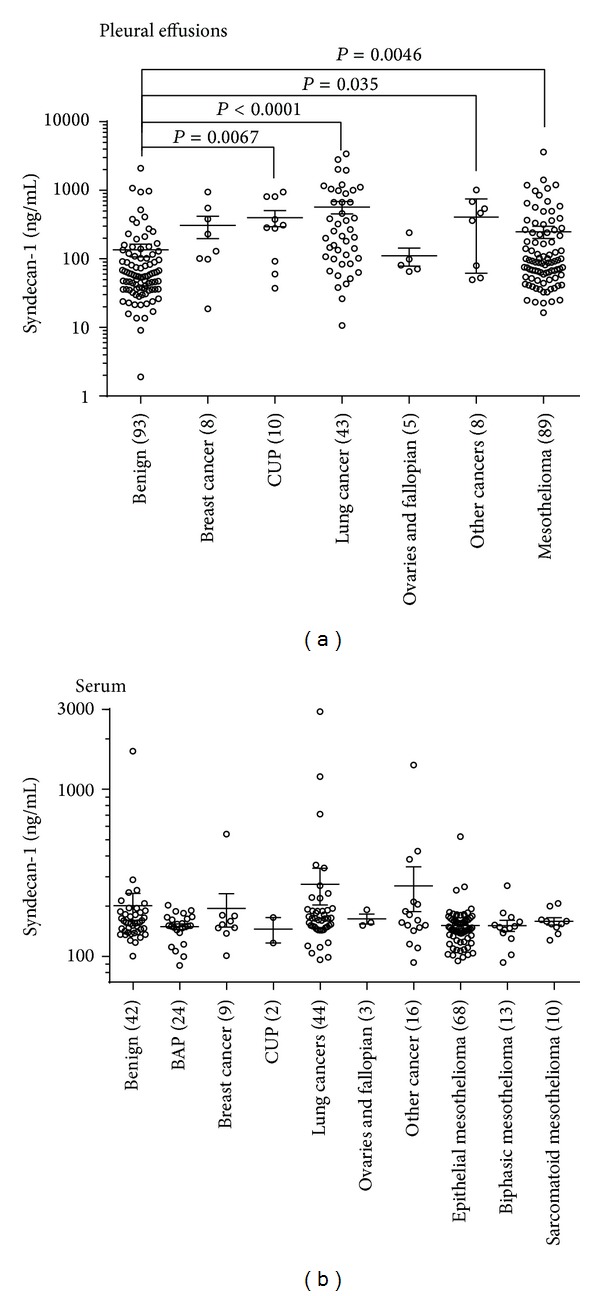
Soluble syndecan-1 levels in various malignant tumours. Significantly elevated soluble syndecan-1 levels were measured in several malignant tumours compared to benign disease. *P* values are derived from Kruskal-Wallis tests, with Dunn's* post hoc* tests. Benign asbestos pleuritis (BAP) and cancer of unknown primary (CUP). Where no *P* values are stated, it indicates a nonsignificant association. Lines represent mean and error bars represent standard error of the mean (SEM).

Syndecan-1 and osteopontin levels were both significantly higher in malignant pleural effusions than in those with benign conditions, the difference being more pronounced with syndecan-1. However, neither soluble syndecan-1 nor osteopontin differentiated patient groups in sera (Figure 1). The pleural effusion levels of syndecan-1 were highest in carcinomas, although patients diagnosed with malignant mesothelioma also had significantly elevated levels compared to benign disease (Figure 2). Paired pleural effusion and serum samples showed moderate correlation of soluble syndecan-1 (*r* = 0.45, *P* = 0.05). Furthermore, syndecan-1 and osteopontin levels correlated in both pleural effusions (*r* = 0.18, *P* = 0.019) and sera (*r* = 0.25, *P* < 0.0001; see Supplementary Figure S1 in Supplementary Material available online at http://dx.doi.org/10.1155/2014/419853). ELISA results for syndecan-1 and osteopontin of all patients can be downloaded from the Dryad online repository (doi: 10.5061/dryad.c42t7).

### 3.2. Diagnostic Performance of Syndecan-1 and Osteopontin

**Figure 3:**
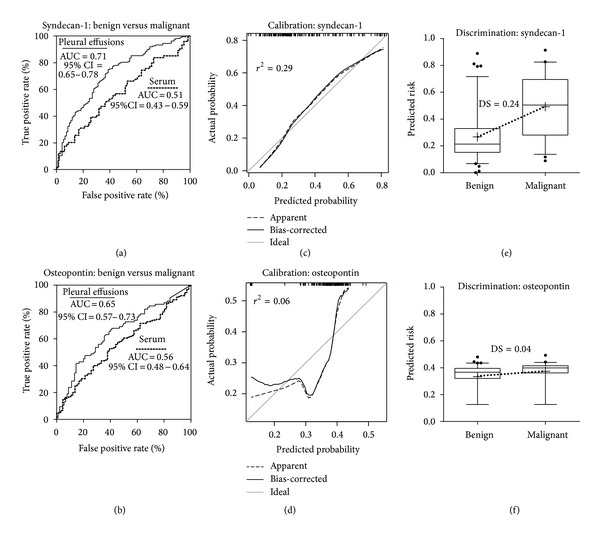
Soluble syndecan-1 has a diagnostic value for malignancy in pleural effusions. ROC plots for syndecan-1 in pleural effusions and serum (a) and osteopontin in pleural effusions and serum (b). Figures (c) to (f) only pertain to pleural effusions. Calibration curves evaluate the relation between the predicted and actual probabilities for soluble syndecan-1 and osteopontin, panels (c) and (d), respectively. Nagelkerke's *r* ^2^ is displayed in Figures (c) and (d). Box plots of predicted risks show a greater discrimination between malignant and benign effusions when using syndecan-1 as opposed to osteopontin (e, f). Discrimination slopes (DS; dotted lines) are calculated as the difference between mean predicted risks (+). Boxes show median and interquartile ranges, while whiskers represent the 5th to 95th percentile. Outliers are presented as black dots.

Syndecan-1 levels in pleural effusions significantly predicted malignant disease (odds ratio 8.59, 95% CI 3.67 to 20.09). Area under the ROC curve was 0.71 (95% CI 0.65, 0.78), model calibration was acceptable (Nagelkerke's *r* ^2^ = 0.29), and goodness of fit was good (*P* = 0.99). Discrimination slope was 0.24 (Figures 3(a), 3(c), and 3(e)). Syndecan-1 had a sensitivity of 74.9% and specificity of 61.3% at the cutoff of 65.7 ng/mL (Table 2). However, in sera, syndecan-1 showed poor prediction of a malignant disease (odds ratio 1.31, 95% CI 0.26 to 6.65; AUC 0.51, 95% CI 0.43 to 0.59 (Figure 3(a)); Nagelkerke's *r* ^2^ < 0.01; goodness of fit = 0.54; discrimination slope < 0.01).

Osteopontin in effusions also significantly predicted a malignant disease (odds ratio 1.39, 95% CI 1.03, 1.88). Area under the ROC curve was 0.65 (95% CI 0.57 to 0.73), the model calibration was poor (Nagelkerke's *r* ^2^ = 0.06), and goodness of fit was poor although not below the usual 0.05 threshold for rejection (*P* = 0.09). Discrimination slope was 0.04 (Figures 3(b), 3(d), and 3(f)). Osteopontin had a sensitivity of 65.5% and specificity of 61.1% at the cutoff of 2034.0 ng/mL. Sensitivities, specificities, positive predictive values, and negative predictive values are represented in Table 2. Serum osteopontin also showed poor prediction for a malignant disease (odds ratio 1.99, 95% CI 0.87 to 4.58; AUC 0.56, 95% CI 0.48 to 0.64 (Figure 3(b)); Nagelkerke's *r* ^2^ = 0.02; goodness of fit = 0.58; discrimination slope = 0.01).

### 3.3. Prognostic Value of Soluble Syndecan-1 and Osteopontin

**Figure 4:**
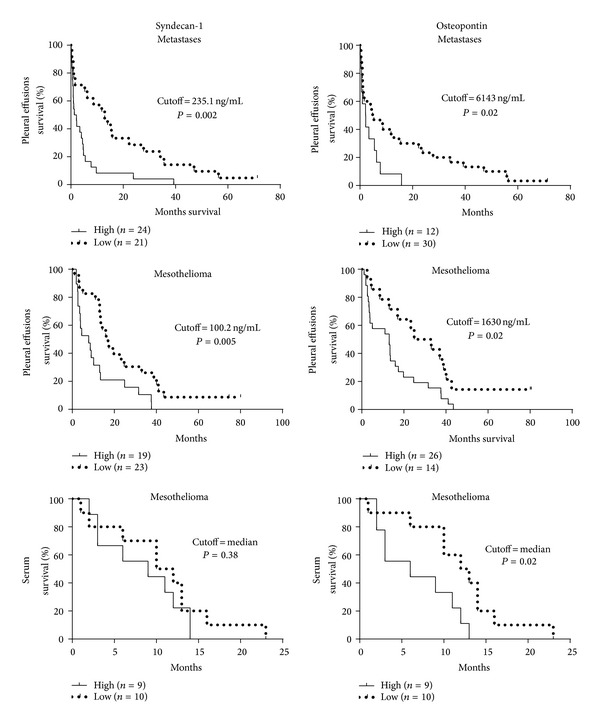
Both soluble syndecan-1 and osteopontin have prognostic roles for patients with malignant mesothelioma or metastatic pleural disease. Cutoffs for “high” and “low” syndecan-1 expression were identified by the online web application Cutoff Finder [42]. The effect of each marker on a time-to-event outcome is presented in corresponding Kaplan-Meier plots. *P* values are based on log-rank (Mantel-Cox) tests.

Cutoff values derived from highest and most significant hazard ratios for pleural effusions are reported in Supplementary Figure S2. Median survival time of patients with pleural metastases and an effusion syndecan-1 level <235.1 ng/mL was 12.9 months, compared to only 1.7 months in patients with higher syndecan-1 levels (hazard ratio 2.38, 95% CI 1.56 to 5.44). Stratifying malignant mesothelioma patients in “low” and “high” syndecan-1 level using a cutoff of 100.2 ng/mL resulted in median survival times of 17.0 and 7.8 months, respectively (hazard ratio 2.77, 95% CI 1.35 to 5.68; Figure 4). Pleural effusion osteopontin levels also predicted survival time; at the cutoff level of 6.14 *μ*g/mL, the median survival differed from 5.1 to 2.2 months for patients with metastatic pleural disease between “low” and “high” expression, respectively (hazard ratio 2.05, 95% CI 1.19 to 6.12). For patients with malignant mesothelioma, the effusion osteopontin cutoff of 1.6 *μ*g/mL resulted in median survival times of 29.0 and 13.0 months, for “low” and “high” expression, respectively (hazard ratio 2.16, 95% CI 1.16 to 4.15; Figure 4).

Among mesothelioma patients with serum syndecan-1 levels higher than the median (144 ng/mL), the median survival time was 9.0 months, while in those with lower serum levels, it was 11.0 months; however, this difference was not statistically significant (hazard ratio 1.43, 95% CI 0.63 to 3.98; Figure 4). The median osteopontin level in serum (185 ng/mL) separated mesothelioma patients in those with “low” and “high” expression, showing median survival times of 12.5 and 6.0 months, respectively (hazard ratio 2.45, 95% CI 1.36 to 10.32; Figure 4). There was no significant difference in VEGF, age, or gender between any of the analysed groups of “low” and “high” expression levels (Mann-Whitney test; data not shown).

### 3.4. Relation between Cell-Bound and Soluble Syndecan-1

**Figure 5:**
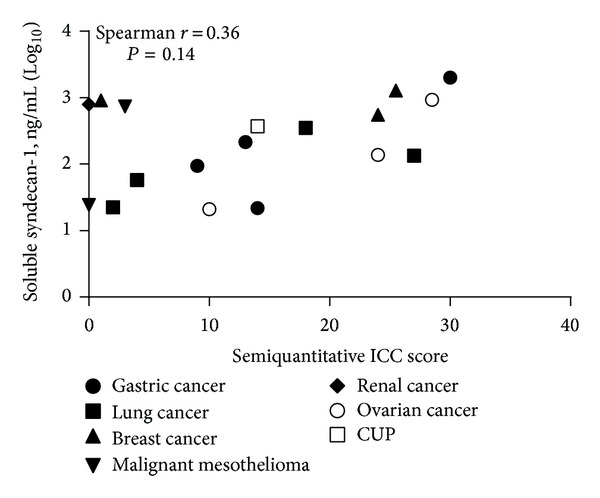
Strong cellular immune-reactivity for syndecan-1 on malignant cells correlates with higher levels of soluble syndecan-1 in pleural effusions. Spearman correlation was used to test the goodness of fit between levels of soluble syndecan-1 and cell bound syndecan-1 intensity times the percentage of positive tumour cells (semiquantitative ICC score; *r* = 0.36; *P* = 0.14). ICC = immunocytochemistry.

To assess the relationship between cell-bound and soluble syndecan-1, we performed immunocytochemistry on cells in pleural effusion samples paired with their ELISA readout of soluble syndecan-1 levels (*n* = 18). Linear regression of staining intensity times percentage syndecan-1 reactive tumour cells (semiquantitative ICC score) and pleural effusion levels indicate a positive correlation (*r* = 0.36, *P* = 0.14; Figure 5).

## 4. Discussion

The importance of the syndecan proteoglycan family has been implicated in several aspects of cancer [20–22, 25, 43–45]. Particularly in epithelial cancers, syndecan-1 has been associated with angiogenesis, invasion, proliferation, differentiation as well as diagnosis, and patient survival [23–25, 30, 46–48]. In malignant mesothelioma, Kumar-Singh et al. have shown that the membrane associated form of syndecan-1 is a good prognostic factor, where high tissue levels of syndecan-1 correlate with longer survival [32]. Furthermore, syndecan-1 has higher expression in epithelioid cell lines as compared to sarcomatoid cell lines [32], a phenotypic correlation that can explain part of the prognostic findings. Overexpression of syndecan-1 induces epithelial morphology and inhibits proliferation in mesothelioma cell lines [49], indicating a syndecan-1 dependent differentiation along the epithelial mesenchymal transition axis in this cancer. In this study we analyse the role of soluble syndecan-1 for its diagnostic and prognostic importance in pleural effusions and serum from patients with malignant diseases. The levels of shed syndecan-1 were elevated in pleural effusions from both metastatic carcinomas and malignant mesothelioma compared to patients with nonmalignant disease. As demonstrated by higher odds ratios, increased AUCs, better calibration, and better discrimination, soluble syndecan-1 had better diagnostic accuracy for a pleural malignancy compared to the reference biomarker osteopontin. The obtained AUCs indicate that effusion contents of these two compounds could be of diagnostic use if combined with other biomarkers in a battery, while their importance in serum seems to be more limited.

Stratifying the patients into disease subgroups, the highest levels were seen in effusions due to metastases from lung cancer, breast cancer, and gastric cancer, possibly reflecting their epithelial origin. Syndecan-1 levels were also higher in effusions from patients with mesothelioma. This suggests a more general pathophysiological role of syndecan-1 in cancers.

Effusion and serum levels of syndecan-1 correlated moderately, but the diagnostic value of syndecan-1 could not be demonstrated in sera. Here the differences between analysed patient groups are smaller, as is the dynamic range* within* groups. Pleural effusions have been shown to carry higher diagnostic accuracy than serum for several malignant mesothelioma biomarkers, a phenomenon that has been attributed to less interference by liver metabolism or elimination by the kidneys [50, 51]. The lack of diagnostic value in serum could also be explained, in part, by the contribution from other body fluids into this medium.

We further show that soluble syndecan-1 in pleural effusions carry strong prognostic value for patients with pleural malignancies. Low levels of shed syndecan-1 predict a more favourable prognosis, showing more than 11 months increased median survival for patients with pleural metastases and 9.2 months for malignant mesothelioma; both are diseases with average median survival times of around, or less than, one year. This trend is also seen in the two main subgroups of metastatic tumours—that is, lung and breast adenocarcinoma—although both these subgroups are too small to show statistical significance. These findings are in concordance with earlier studies on multiple myeloma [34] and lung cancer [35], in both elevated levels of syndecan-1 in sera predicted poor prognosis. There are several possible explanations for the prognostic properties of syndecan-1. In the malignant cell, syndecan-1 participates in the regulation of basic functions such as proliferation and cell migration, thereby possibly influencing the cancer progression and ultimately patient survival. One caveat to keep in mind is that although all pleural metastases represent advanced stages, it could still be that the increase in soluble syndecan-1 merely reflects tumour burden and thereby can be linked to shorter survival.

Structurally, the extracellular domain of syndecan-1 carries attachment sites for heparan sulfate and chondroitin sulfate chains. Since heparan sulfate chains act as binding sites for several ligands such as growth factors and chemokines, proteolytic shedding of the extracellular domain is crucial in regulating various signaling pathways [52–54].

In our cohorts, higher levels of soluble syndecan-1 corresponded to stronger immunocytochemical staining of syndecan-1. It seems that the soluble fraction increased proportionally to the expression of syndecan-1 on these cells. We cannot, however, exclude a concomitant increase in protease activity and syndecan-1 ectodomain shedding in the same cells. Such shedding has been associated with cancer progression, risk for recurrence, and prognosis [27, 28, 55–57]. It can, however, not be excluded that some of the released syndecan-1 is the result of cellular lysis or tumour derived exosomes containing this proteoglycan [58].

Several studies suggest elevated pretreatment soluble serum syndecan-1 level as a predictor of poor prognosis and impaired effect of chemotherapy [59, 60]. Furthermore, low serum syndecan-1 level can predict sensitivity to anticancer therapy in larynx and hypopharynx cancer, whereas high posttreatment level is an indicator of relapse [61].

In this study we show a possible diagnostic and prognostic role for soluble syndecan-1 in effusion cytology. The use of this proteoglycan as an effusion biomarker could be helpful in the evaluation of effusions. Similar to most established biomarkers—immunological or immunocytochemical—the discriminatory power of this analysis is insufficient to use as a sole diagnostic marker in the individual case. The combination of this parameter with other biomarkers in logistic models remains to be studied. This, however, should then warrant further studies on the mechanisms behind the release of syndecan-1 as well as possible predictive effects and effects on epithelial mesenchymal transition in tumours.

## 5. Conclusion

In summary, this study describes the clinical value of analysing soluble syndecan-1 in serum and pleural effusions. Syndecan-1 separates malignant and benign conditions when measured in pleural effusion supernatants. Furthermore, we show a striking correlation between syndecan-1 levels and patients' survival, which is of interest for future predictive translational research. Hence, the inclusion of measuring syndecan-1 could be a valuable clinical tool, whether on its own or in a panel of soluble biomarkers.

## Supplementary Material

Figure S1: The correlation between syndecan-1 and osteopontin in pleural effusions and serum.
Figure S2: Continuous hazard ratios of syndecan-1 and osteopontin for the determination of optimal survival curves.
Figure S3: The diagnostic value of syndecan-1 in patient subgroups.

## Figures and Tables

**Table 1:** Demographic data.

Pleural effusions	Number of patients	Male (%)	Female (%)	Age, median (IQR)
Benign	93	63 (68)	30 (32)	68 (54–80)
Malignant	74	23 (31)	51 (69)	68 (62–78)
Lung cancer	*43 *	*14 (33) *	*29 (67) *	*70 (62–79) *
Breast cancer	*8 *	*1 (12) *	*7 (88) *	*64 (47–80) *
Ovarian and fallopian cancers	*5 *	*0 (0) *	*5 (100) *	*70 (66–78) *
Other malignancies	*8 *	*3 (37) *	*5 (63) *	*65 (63–77) *
Cancer of unknown primary	*10 *	*5 (50) *	*5 (50) *	*68 (52–80) *
Malignant mesothelioma	89	79 (89)	10 (11)	70 (63–78)

Sera	Number of patients	Male (%)	Female (%)	Age, median (IQR)

Benign	66	52 (79)	14 (21)	59 (48–71)
Benign asbestos pleuritis	*24 *	*23 (96) *	*1 (4) *	*62 (54–73) *
Malignant	74	44 (59)	30 (41)	61 (54–69)
Lung cancer	*44 *	*34 (77) *	*10 (23) *	*63 (57–70) *
Breast cancer	*9 *	*1 (11) *	*8 (89) *	*56 (48–71) *
Ovarian and fallopian cancers	*3 *	*0 (0) *	*3 (100) *	*57 (45–64) *
Other malignancies	*16 *	*9 (56) *	*7 (44) *	*62 (52–72) *
Cancer of unknown primary	*2 *	*1 (50) *	*1 (50) *	*56 (50–61) *
Malignant mesothelioma	91	34 (37)	57 (63)	65 (56–69)
Epithelioid	*62 *	*23 (37) *	*39 (63) *	*65 (54–71) *
Biphasic	*13 *	*4 (31) *	*9 (69) *	*57 (55–69) *
Sarcomatoid	*10 *	*8 (80) *	*2 (20) *	*63 (61–65) *
Undetermined	*6 *	*2 (33) *	*4 (66) *	*64 (55–70) *

Age (IQR: interquartile range) and patient subgrouping in the two analysed materials. The high proportion of female mesothelioma patients in the serum material is most likely due to environmental asbestos and erionite exposure, which relates to geographical distribution and has also been reported by others [36].

**Table 2:** Cut-offs were derived from the maximum sensitivity × specificity in respective receiver operator characteristic curve.

Biomarker	Fluid	Cut-off (ng/mL)	Sensitivity (%)	Specificity (%)	PPV (%)	NPV (%)
Syndecan-1	Pl. effs	65.7	74.9	61.3	0.77	0.57
Syndecan-1	Sera	151.6	46.1	59.1	0.70	0.27
Osteopontin	Pl. effs	2034.0	65.5	61.1	0.60	0.65
Osteopontin	Sera	217.3	52.7	60.6	0.77	0.34

Pl. effs: pleural effusions; PPV: positive predictive value; NPV: negative predictive value.
