# Supplementary material for: Diagnostic and Prognostic Value of Soluble Syndecan-1 in Pleural Malignancies
Source: Biomed Res Int. 2014 Jul 24;2014:419853. doi: 10.1155/2014/419853 (PMC4131558; doi:10.1155/2014/419853)
Supplement: Supplementary file 1 — Figure S1: The correlation between syndecan-1 and osteopontin in pleural effusions and serum. Figure S2: Continuous hazard ratios of syndecan-1 and osteopontin for the determination of optimal survival curves. Figure S3: The diagnostic value of syndecan-1 in patient subgroups. [file 419853.f1.pdf]

Supplementary material

This file has been provided by Mundt F, et al. to give supplementary information about their study:

Diagnostic and prognostic value of soluble syndecan-1 for pleural metastases and malignant pleural mesothelioma

Index:

Figure S1, page 1
Figure S2, page 2
Figure S3, page 3

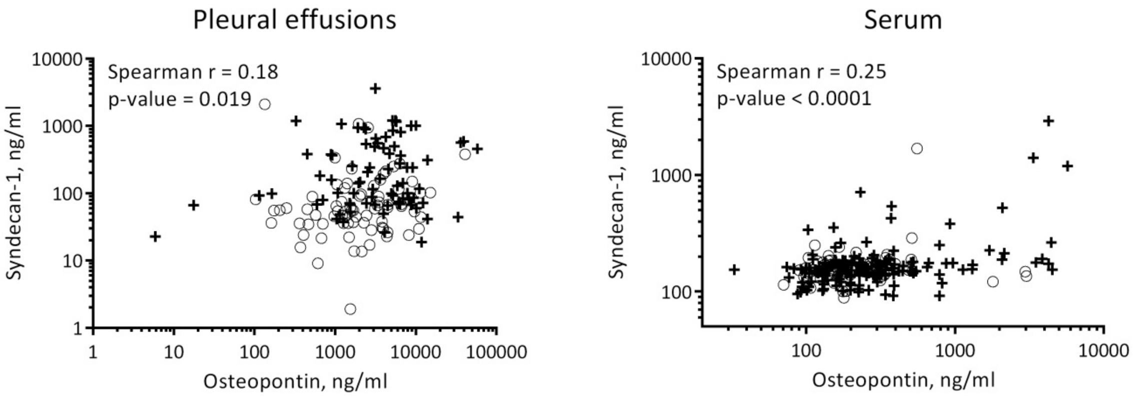

Figure S1. The correlation between syndecan-1 and osteopontin in pleural effusions and serum. Pluses (+) denote malignant cases, while circles denote patients with benign disease.

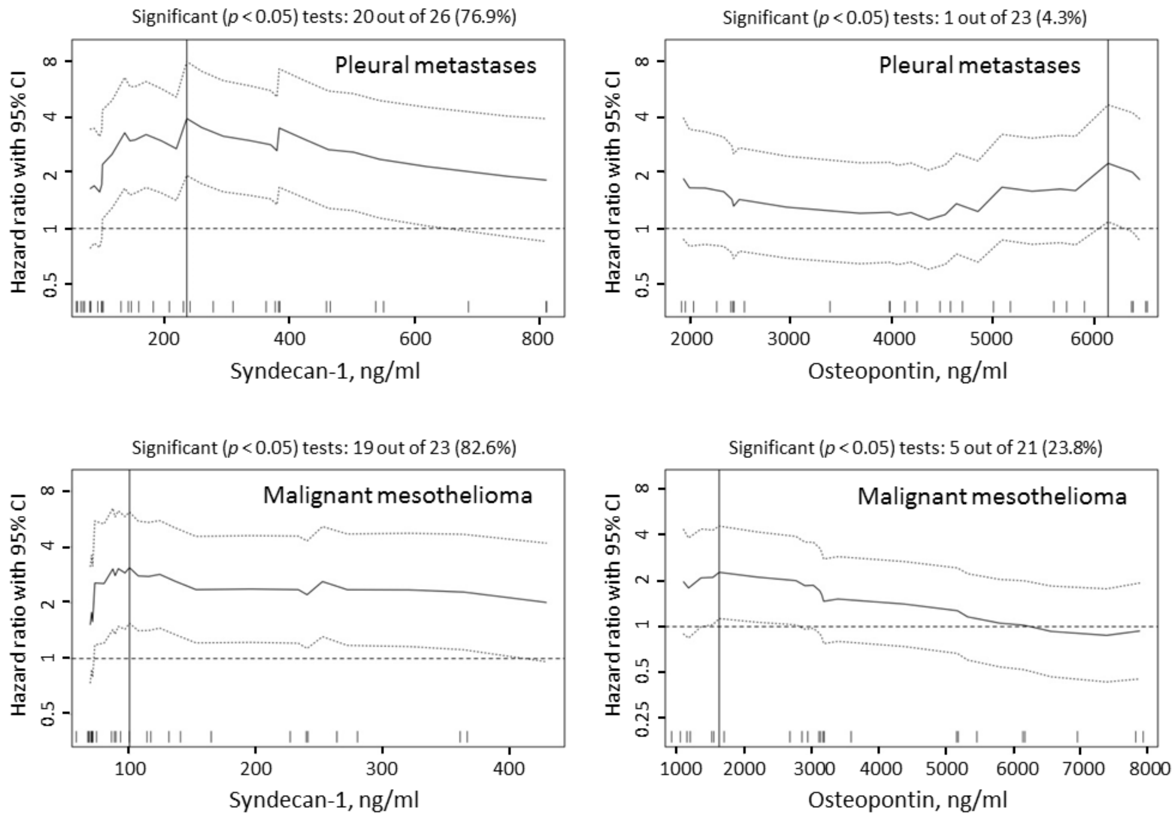

Figure S2. Hazard ratio and 95% confidence interval (95% CI; dotted lines), plotted over a continuum of either syndecan-1 or osteopontin concentrations. Calculations and graphs were created using the *Cutoff Finder* application (http://molpath.charite.de/cutoff/). Highest and most significant hazard ratio (vertical solid line) was subsequently chosen as cut-offs for Kaplan-Meier estimates.

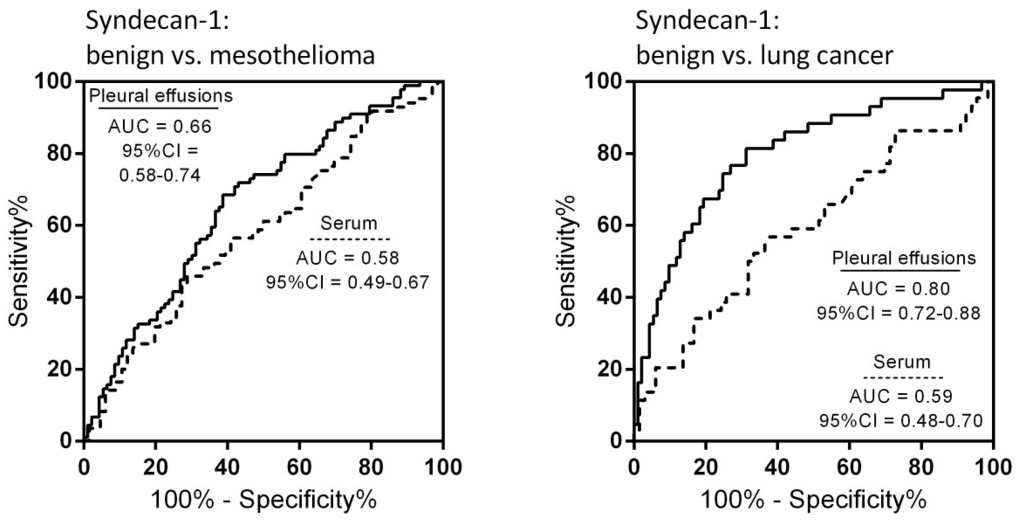

Figure S3. Receiver operating characteristic curves of subgroups showing syndecan-1's diagnostic value in pleural effusions or serum. Enough patients for statistically powerful analysis were present when comparing benign cases to patients with either malignant pleural mesothelioma or lung adenocarcinoma.
